# Advances in Computational Methods for Modeling Photocatalytic Reactions: A Review of Recent Developments

**DOI:** 10.3390/ma17092119

**Published:** 2024-04-30

**Authors:** Sergey Gusarov

**Affiliations:** Digital Technologies Research Centre, National Research Council Canada, Ottawa, ON K1A 0R6, Canada; sergey.gusarov@nrc-cnrc.gc.ca

**Keywords:** DFT, DFTB, multiscale, machine learning, quantum computing, photocatalysis

## Abstract

Photocatalysis is a fascinating process in which a photocatalyst plays a pivotal role in driving a chemical reaction when exposed to light. Its capacity to harness light energy triggers a cascade of reactions that lead to the formation of intermediate compounds, culminating in the desired final product(s). The essence of this process is the interaction between the photocatalyst’s excited state and its specific interactions with reactants, resulting in the creation of intermediates. The process’s appeal is further enhanced by its cyclic nature—the photocatalyst is rejuvenated after each cycle, ensuring ongoing and sustainable catalytic action. Nevertheless, comprehending the photocatalytic process through the modeling of photoactive materials and molecular devices demands advanced computational techniques founded on effective quantum chemistry methods, multiscale modeling, and machine learning. This review analyzes contemporary theoretical methods, spanning a range of lengths and accuracy scales, and assesses the strengths and limitations of these methods. It also explores the future challenges in modeling complex nano-photocatalysts, underscoring the necessity of integrating various methods hierarchically to optimize resource distribution across different scales. Additionally, the discussion includes the role of excited state chemistry, a crucial element in understanding photocatalysis.

## 1. Introduction

Photocatalysis is a dynamic research domain dedicated to optimizing and controlling chemical reactions utilizing radiant energy, specifically light. The acceleration of these reactions occurs either through direct interaction with light or by inducing excitation in a substance that catalytically facilitates the primary reaction [1,2]. This evolving field exhibits substantial potential in addressing challenges related to energy [3] and the environment [4,5,6], leveraging the emulation of intricate processes inherent in natural photochemistry while incorporating sustainable materials.

Photocatalysts are molecular systems such as nanoparticles, surfaces, or organic/inorganic molecules with specific semiconducting features such as light absorption, charge transfer, and electronic and geometrical characteristics. Researchers are actively exploring innovative light-responsive materials and making significant progress in understanding their mechanisms. These materials have the potential to revolutionize the field of photocatalysis by enhancing process efficiency and broadening its applications [7,8]. Photocatalysis offers considerable promise across diverse scientific domains, such as energy, environmental solutions, and advancements in health and materials. Notably, its capacity to harness sunlight to generate hydrogen fuel stands out as a clean and renewable energy source, crucial for reducing pollution and lessening reliance on non-renewable fossil fuels. Beyond energy-related applications, photocatalysis shows a remarkable capacity for light signal detection, paving the way for improved communication and security technologies. By discerning light intensity, wavelength, or color, this technology supports optical communication, imaging, and encryption, thus aiding the progress of information and security systems. Furthermore, photocatalysis significantly contributes to environmental well-being, evident in its capacity to purify water [9] or air [10] from pollutants and pathogens. The capability of photocatalysis directly enhances environmental quality and, subsequently, human health, rendering it a crucial tool in attaining the global objective of clean and safe resources. Another facet of its versatility lies in materials science, where photocatalysis plays an instrumental role in creating innovative compounds and materials, spanning from drugs and polymers to nanomaterials and catalysts [7]. These innovations find applications in various industrial [11] and biomedical fields [12], underscoring the far-reaching impact of photocatalysis.

Moreover, photocatalysis exhibits the capability to alter surface or interface properties, thereby influencing the functionality and performance of devices such as batteries [13], capacitors, and solar cells [14]. This not only contributes to technological advancements but also enhances the devices’ efficiency and sustainability, thereby improving their overall reliability. A particular focus within the field of photocatalysis is on improving solar cells [15]. Researchers aim to enhance the efficiency and stability of converting solar energy into electrical energy, striving to make significant strides in the development of sustainable and efficient energy sources [16]. In summary, photocatalysis emerges as a multifaceted and promising field, well-positioned to address complex global challenges across various scientific and technological domains.

In photocatalysis, computational modeling plays a pivotal role, serving as a foundation for understanding the intricate mechanisms governing photocatalysis and designing semiconductor photocatalyst systems [17]. Employing simulations that capture the dynamics among numerous electrons, nuclei, and molecules within condensed matter [18], computational modeling facilitates an in-depth exploration of atomic and electronic structures, as well as the dependent properties of nanostructures at a sub-nanometer scale [19]. This capability allows researchers to formulate innovative theoretical models for photocatalyst materials and interfaces, indispensable in the strategic design and engineering of semiconductor photocatalyst systems [20,21].

Quantum chemical methods, such as ab initio and semi-empirical approaches, are indispensable in photocatalysis modeling [22,23,24,25,26]. Quantum chemical methods are significant for their ability to intricately capture and represent various chemical properties at the quantum level. Leveraging these methods provides profound insights into the complex processes involved in photocatalysis. With first-principles calculations on high-performance computing platforms, a virtual laboratory can be established to unravel the nuanced interplay between physical properties, like atomic structures, defects, and interfaces, and the electronic structure of materials. This approach proves instrumental in testing novel concepts and ideas for the development of potentially efficient photocatalyst materials and devices. Furthermore, computational modeling guides the design and optimization of metal–organic frameworks (MOFs) for photocatalysis [27,28], emphasizing the tailoring of band edge positions to achieve optimal photocatalytic performance. Additionally, the application of modeling and simulation through computational fluid dynamics (CFD) proves critical in the design and optimization of microreactors employed in photoredox catalysis [29,30]. This computational approach enhances our understanding of reagent interactions and the influence of light within the reaction medium. In summary, computational modeling emerges as an indispensable tool for propelling the field of photocatalysis forward. It not only provides comprehensive insights into the fundamental principles governing light–matter interactions but also facilitates the discovery of novel materials and devices with diverse applications.

Methods in chemical kinetics [31,32] also play a crucial role in photocatalysis, where numerous simultaneous reactions may occur concurrently. These simulations use the energetics of different intermediate compounds that can be calculated from quantum mechanics. The intricacies of photocatalytic reaction kinetics add to the complexity, being contingent on various factors like the catalyst’s nature, the reactants involved, and the intensity of light. External influences such as impurities, temperature, and the pH of the reaction medium can impact the kinetics of photocatalytic reactions. Studying chemical kinetics in the context of photocatalysis is essential for understanding reaction mechanisms and optimizing the performance of photocatalytic systems.

Although conventional computational methods are useful for modeling photocatalytic processes, their effectiveness is limited by the inherent complexity of the reactions under investigation. Overcoming these challenges requires primarily refining the precision and applicability of these methods [33]. Precision plays a crucial role due to the intricate nature of photocatalysis, characterized by complex interactions at the molecular level [34]. Traditional computational methods face limitations in capturing these intricate dynamics, necessitating refinement to accurately represent the interplay among electrons, nuclei, and molecules within photocatalytic systems. At the same time, it is essential to enhance the applicability of computational methods, due to the diverse and dynamic characteristics of photocatalytic processes. Researchers are working to develop methodologies that can accommodate various conditions, materials, and environmental factors, endowing the models with versatility and relevance in diverse real-world scenarios [35,36,37]. In essence, the challenge lies not only in improving accuracy but also in augmenting the adaptability of computational methods to effectively address the varied scenarios encountered in photocatalysis research.

This review concludes by exploring the promising future of modeling in photocatalysis research, highlighting the potential of multiscale modeling approaches [38,39], as well as advancements in machine learning [40,41] and innovative quantum algorithms [42], to overcome existing challenges. In this regard, multiscale modeling serves as a pivotal tool in bridging the divide between various levels of description, effectively capturing interactions and feedback mechanisms. For instance, it seamlessly integrates quantum mechanical calculations of the electronic structure and optical properties of photocatalytic materials with kinetic models of reaction pathways and rates. It also accommodates environmental factors such as temperature, pressure, and solvent effects, offering a comprehensive understanding of photocatalytic processes and guiding the rational design of materials and systems. In turn, machine learning and artificial intelligence contribute significantly to the field by facilitating the discovery of new photocatalytic materials, optimizing reaction conditions, and extracting meaningful patterns from vast and noisy datasets. By leveraging existing experimental and simulation data, predictive models could potentially accelerate material screening and evaluation while identifying key factors influencing photocatalytic activity and selectivity. Quantum computing represents a paradigm shift, offering unparalleled accuracy and speed in solving quantum mechanical equations governing electronic and optical properties. Overcoming the scalability and complexity limitations of classical computing, the quantum paradigm enables the simulation of large, realistic photocatalytic systems while exploring quantum phenomena like entanglement, superposition, and tunneling. By unlocking new potentials beyond traditional tools, quantum computing expands the horizons of photocatalysis research.

While multiscale modeling is currently an extensively utilized technique, ML/AI models are under extensive development, and quantum computing is still in its nascent stages. Despite their varying levels of maturity, these techniques collectively drive advancements in photocatalysis research. By enhancing neatness and coherence, this review explores how modeling methods [43,44] impact research in photocatalysis.

## 2. Basics of Photocatalysis

The fundamental principles underlying photocatalysis encompass the interaction of light with matter, the creation of electron–hole pairs, and the ensuing redox reactions taking place on the photocatalyst surface. The foundation of photocatalytic process modeling is based on band structure theory [45]. This solid-state physics concept elucidates the distribution of electron energy levels within solids. Band theory posits that these energy levels are organized into bands, interspersed with “band gaps”—regions devoid of electron states.

The photocatalytic process is initiated by the generation of an electron–hole pair induced by light (photogenerated exciton) within the catalyst (molecule, nanoparticle, surface, etc.). If photon energy is equal to or greater than the photocatalyst’s bandgap, an electron from the valence band of the catalyst is excited and shifted to the unoccupied conduction band (excited-state conduction-band electron), creating a positive hole (valence-band hole) in the valence band. This separation of charges creates a potential for redox reactions to take place on the photocatalyst surface. The electrons in the conduction band can be transferred to an electron acceptor (1), while the hole can oxidize a donor molecule or reduce an oxidant (2):

(1) $$e^{-}+A\to A^{-}\cdot$$

(2) $$h^{+}+H\cdot D\to H^{+}+D$$

This process produces highly reactive intermediate radicals engaging in reactions with reactant molecules present on the photocatalyst surface, leading to the formation of the desired products [46,47,48]. The efficiency of photocatalytic processes is influenced by the band gap of the material. A smaller band gap allows for the absorption of a broader spectrum of light, but it must be large enough to provide the energy needed for the reactions. Bandgap engineering is a strategy used to optimize the band gap for better light absorption and charge carrier dynamics [49].

The water splitting (3) [50,51]

(3) H2O→hν2H2+12O2

(4) $$2H^{+}+2e^{-}\to 2H_{2}$$

(5) H2O+2h+→2H++12O2

and reduction of carbon dioxide (6) [52] are the most well-known photocatalytic processes that are currently under strong research and development.

(6) $$CO_{2}+2H\overset{h\nu }{\to }2H_{2}+O_{2}$$

(7) $$CO_{2}+2H\overset{h\nu }{\to }CO+H_{2}O$$

(8) $$CO_{2}+4H\overset{h\nu }{\to }C+2H_{2}O$$

Water cannot directly absorb sunlight in the first reaction [53] due to its transparency across the entire spectrum. The mechanism through which photon energy is transferred to water molecules entails the initial absorption of sunlight by catalyst, followed by its transfer to H_2_O [54], concluding in a four-step process: first, the absorption of a photon with energy greater than the band gap of the photocatalyst, resulting in the generation of an electron–hole pair; second, the separation of the photoexcited electron and hole; third, their subsequent reaction with a water molecule, yielding hydrogen (reduction, (4)) and oxygen (oxidation, (5)); and finally, the release of the produced hydrogen and oxygen from the surface of the photocatalyst. To efficiently split water, the photocatalyst must have a wide band gap (>1.23 eV). However, in practice, factors such as internal material resistance and the overpotential of the water-splitting reaction elevate the necessary bandgap energy, ranging from 1.6 to 2.4 eV [55,56]. It is also crucial to emphasize that water splitting is an energy-demanding process with ΔH > 0. Titanium dioxide is an example of a semiconductor with the right band structure. Its bandgap is 3.2 eV, which means it can absorb ultraviolet light and generate electron–hole pairs. By irradiating TiO_2_ with light, electrons are excited from the valence band to the conduction band, leaving positive holes behind. After migrating to the surface of TiO_2_, electrons and holes can react with water molecules [57]. Natural photosynthesis occurs in plants and some bacteria and splitting occurs because of the absorption of the energy from four photons, which is then transformed into chemical energy through a complex biochemical pathway [58]. This process requires higher energy input from photons, making it more challenging to achieve compared to photochemical splitting.

The second reaction is a multistep process that involves various intermediates and products depending on catalyst properties and reaction conditions (6-8). The reaction pathways and mechanisms of CO_2_ reduction are influenced by factors such as the catalyst material, structure, morphology, composition, surface area, and defect density, as well as the applied potential, pH, temperature, pressure, solvent, electrolyte, and gas diffusion [52,59]. For such reactions, it is very convenient to analyze the energy diagrams for possible intermediates, as this allows for the prediction of the most promising pathway among the number of parallel processes [60]. Catalytic surface modifications, such as defects, vacancies, and additions, can also be used to optimize and control the reaction. As a result, the electronic structure, adsorption properties, and catalytic activity of catalysts can be altered [61].

In general, the photocatalytic reaction process can be broadly split into the following major steps [62,63,64]:

1. Adsorption: The reactant molecule is adsorbed onto the semiconductor surface, creating a physical or chemical bond with the catalyst surface, which facilitates further interactions. This adsorption process is influenced by factors such as the catalyst’s surface area, charge, and affinity.
2. Exciton(s) formation: The catalyst absorbs light within a specific wavelength range, typically in the UV or visible range, and then generates electron–hole pairs through a process known as exciton formation. The excited electrons transition from the valence band to the conduction band, leaving positive holes in the former. These electron–hole pairs, or excitons, exhibit high reactivity and a comparably short lifetime.
3. Reaction: The electrons and/or holes react with the adsorbed molecule, either directly or indirectly. Direct reactions involve the transfer of electrons or holes from the catalyst to the molecule, resulting in oxidation or reduction. Indirect reactions involve the generation of reactive oxygen species (ROS), such as hydroxyl radicals, superoxide anions, or hydrogen peroxide, from the reaction of electrons or holes with water or oxygen. The ROS then attack the adsorbed molecule, causing its degradation or mineralization.
4. Over-reaction: The reaction between the electrons or holes and the adsorbed molecule continues until the molecule is completely broken down into simple and harmless products, such as water, carbon dioxide, or inorganic ions. This process, also known as mineralization or complete oxidation, guarantees that no toxic intermediates remain in the solution.
5. Desorption: The final step is the release of the reaction products from the surface, which frees up the active sites on the catalyst for new adsorption and reaction cycles. Desorption is influenced by factors such as the concentration of reactants, temperature, and pH.

These steps are crucial for the overall effectiveness of photocatalysis. By leveraging these mechanisms, photocatalysis has emerged as an essential technology for various applications, including environmental remediation [65], energy production, and chemical synthesis [66].

In recent years, significant progress has been made in the field of photocatalysis. There have been efforts to modify the structure of photocatalysts to enhance their performance and efficiency. Studies have focused on exploring new materials for photocatalysts, including perovskites [67,68], metal–organic frameworks [69], and nanomaterials [70]. The use of hybrid systems, such as coupling photocatalysts with electrocatalysts [71] or photo-electrocatalysts, has also been explored to enhance overall performance.

## 3. Quantum Chemical Methods in Photocatalysis: Ab Initio, DFT, Semi-Empirical

Ab initio quantum chemical methods provide valuable advantages in understanding and enhancing photocatalysis processes. These methods enable the analysis of precursor characteristics, such as catalyst bandgaps, the density of states, and adsorption spectra, offering initial information for subsequent machine learning as well as facilitating a detailed examination of the entire process [72,73]. These methods involve solving the electronic Schrödinger equation from first principles, without relying on experimental data. There are several ways in which ab initio quantum chemical methods contribute to advancing the understanding of photocatalysis:

- Detailed electronic structure analysis: These methods provide an accurate description of the electronic structure of molecules involved in photocatalysis, aiding in the prediction of absorption energy, charge transfer, and the dynamics of intermediate compounds during photochemical reaction [74,75,76].
- Mechanistic insight: Modeling can offer insight into the step-by-step mechanisms of photocatalytic reactions, including light absorption, charge transfer, and bond breaking, offering a better understanding of the complex processes involved [24,77,78,79,80].
- Prediction of excited states: Ab initio methods can predict the properties of excited electronic states, which are crucial for understanding the conversion of light energy into chemical reactions [81,82,83].
- Screening and design of catalysts: By calculating properties like reaction barriers and energetics, ab initio methods help identify optimal catalyst structures for enhancing photocatalytic activity and efficiency [84,85,86].
- Quantitative prediction of reaction rates: First principles methods combined with kinetic models enable the prediction of reaction rates, aiding in the design of photocatalysts with improved performance [87].
- Insights into reaction mechanism dynamics: Ab initio molecular dynamics simulations provide real-time insights into the movement of atoms and electrons during photocatalytic reactions, offering a dynamic perspective of the processes involved [88,89,90].
- Tailoring material properties: These methods aid in optimizing the properties of materials used in photocatalysis, such as band gaps and surface reactivity, leading to the design of materials that efficiently promote desired photochemical reactions [91,92,93,94].

Eventually, ab initio modeling enables the rational design of photocatalysts with enhanced efficiency and selectivity. This is achieved by providing a comprehensive understanding of the reaction mechanisms, guiding catalyst selection, and offering a complete picture of their electronic and dynamic properties. Quantum chemistry provides a rich toolbox of computational methods for modeling photocatalytic systems and processes, advancing the development of sustainable energy and environmental solutions. These methods encompass a range of phenomena, including chemical reactions, excited state dynamics, and light absorption and emission. However, modeling photocatalytic processes poses significant challenges due to their complex nature and multiscale character. Wave function (WF) methods, while accurate and powerful, are often impractical for such calculations due to their high computational cost and low scalability for large and complex systems. As a result, methods that utilize electronic density functional theory (DFT) representation have become more favorable alternatives. Nonetheless, caution must be exercised when using these methods, as modern density functional theory functionals, though widely used, are approximate and may be tailored to specific molecular systems and processes.

In its simplest, time-independent form, the Schrödinger (or Kohn–Sham) equation has various solutions based on different methods and approximations. These methods can be initially categorized into variational- and perturbation-type methods [95,96] and their specificity determines their applicability to studying photocatalysis. Variational methods are based on the principle of minimizing the energy of a trial wave function (in the case of DFT, represented in one determinant form), which approximates the system’s true wave function. Perturbation methods, on the other hand, are based on the assumption that the system’s Hamiltonian can be divided into a solvable part and a perturbation term, treated as a minor correction to the solvable part. In photocatalysis, variational methods are often used to study the electronic structure and properties of photoactive materials, such as absorption spectra, excited states, and reaction pathways.

Variational methods offer valuable insights into the electronic structure and properties of photoactive materials, including aspects like excited states and environmental effects on photoinduced processes, which are challenging to discern experimentally. Perturbation methods, on the other hand, are often used to study the effects of external perturbations, such as electric fields, magnetic fields, and light, on the electronic structure and properties of molecules. Perturbation methods can provide insights into the response of molecules to external stimuli, such as the changes in the absorption spectra and the excited states induced by light.

In photocatalysis modeling, the variational approach is a powerful tool for approximating the ground state of a quantum system; in some cases, it could be also used to calculate the excited states. The wave function, which serves as a mathematical expression of the quantum state of the system with parameters to be optimized, can be represented in various forms. The key distinction between these forms lies in the number of configurations employed and their specific representation. The simplest form is the single-configuration wave function, used within methods such as Hartree–Fock and various variants of DFT [97]. In these methods, the wave function is approximated by a single Slater determinant which represents a specific configuration of electrons. However, there are numerous cases where the single-configuration approximation falls short in providing an accurate representation of electron correlation effects, electron transfer, and other critical properties and processes that play an essential role in photocatalysis. This limitation arises because the single-configuration approximation cannot capture the complex, multi-electronic phenomena that are integral to these processes. Consequently, this can lead to significant inconsistencies when predicting observables that are sensitive to these effects, such as reaction rates in photocatalytic processes. For example, conducting computational analysis on metal–organic frameworks (MOFs), which are commonly employed in photocatalytic applications [98,99], presents a considerable challenge [100,101,102]. This complexity arises from the presence of transition metals that serve as active sites within their architecture. These metals can lead to nearly degenerate electron configurations, thereby imparting a multiconfigurational nature to the wave functions of the corresponding excited states. There is a group of methods in which the molecular wave function is represented as a combination of different molecular configurations, which are then optimized [103,104,105]. The simplest of these methods is the configuration interaction (CI) approach, which utilizes a predefined set of orbitals, and configurational coefficients are obtained through variational procedures. On the other hand, the complete active space self-consistent field (CASSCF) method is a multi-configurational approach that simultaneously optimizes both the orbitals’ and CI coefficients’ self-consistency, resulting in the generation of a full CI wavefunction within the selected active space. It is especially useful for analyzing complex chemical systems that have either multi-reference character or non-dynamical/static/strong correlation, such as a photocatalytic metal–organic framework with tunable optical properties [106]. However, the CASSCF method is computationally demanding for large systems, so some further approximations are employed to enhance its efficiency, such as restricting the active space [107], using the density matrix renormalization group (DMRG) technique [108], etc.

The coupled cluster (CC) method [109] is another approach to consider the impact of electron correlations on a system through the systematic inclusion of clusters of excitations while referencing a Hartree–Fock (HF) function. The accuracy of the method notably improves with the incorporation of higher-order excitations, spanning from CCSD (coupled cluster singles and doubles) to CCSDT (coupled cluster singles, doubles, and triples) and beyond, thus leading to a more realistic depiction of the quantum system. While the CC method can be computationally expensive, it has immense potential benefits in studying energetics and reaction pathways [110]. Research efforts have aimed to improve its computational efficiency, and one promising technique is the resolution of identity method [111], which significantly enhances the scalability of the approach.

Perturbation theory (PT)-based techniques are highly favored for incorporating the effect of electronic correlation into low-level approximations and modeling the response of molecular systems to external perturbations like light radiation. However, even the simplest post-Hartree–Fock method, Møller–Plesset Perturbation Theory (MP2), is not commonly used for medium and large systems due to its computational complexity. Nevertheless, MP2 can accurately predict fundamental properties including electronic structure, dipole moment, vibration frequencies, and polarizability for specific molecular systems, such as C–NO_2_ clusters. These properties are crucial in determining the optical response of molecules in the excited state, such as fluorescence decay and second harmonic generation, which are essential for understanding photoinduced processes and excited state dynamics. To incorporate the effects of external fields, MP2 can be integrated with more precise methods like response theory and time-dependent density functional theory (TD-DFT) [112].

Many molecular systems exhibit multiconfigurational character, meaning that their electronic structure cannot be described by a single Slater determinant. This is the case for many organic, inorganic, ligand-field, and conjugated systems which show rich photophysical behavior, such as absorption spectra, excitation energies, potential energy surfaces, and photochemical processes like photoisomerization, photodissociation, and photoreduction. To capture the multiconfigurational nature of these systems, perturbation theory methods can be applied to the wave function obtained via the CASSCF method [113]. This approach is known as complete active space perturbation theory (CASPT2) [114] and is one of the most successful methods for studying the spectroscopy of multiconfigurational systems, despite some theoretical challenges.

Perturbation methods are also widely used to investigate the electronic structure and magnetic properties of transition metal compounds, which exhibit phenomena such as spin-crossover, exchange coupling, and magnetic anisotropy. These phenomena depend on the balance between the electron–electron interactions and the crystal field effects, which can be tuned by PT methods.

Sophisticated time-dependent computational methodologies are necessary for studying the dynamical properties that are crucial to photocatalysis, including charge carrier dynamics and recombination phenomena. One approach is to solve the time-dependent Schrödinger equation, which provides insights into the quantum mechanical behavior of electrons within the material under investigation. Alternatively, computational simulations can be used to trace the temporal evolution of atomic positions and velocities within a molecular framework, accounting for the quantum mechanical effects of the electronic subsystem. These computational techniques, known as ab initio molecular dynamics [115], offer a comprehensive understanding of the photocatalytic process. Moreover, they allow for the incorporation of external variables such as temperature variations, thereby optimizing process parameters for enhanced efficiency.

Solving the exact time-dependent Schrödinger equation for systems with complex Hamiltonians, subject to time-dependent external perturbations, is a formidable computational task. Consequently, a range of methods have been developed to simplify the problem by leveraging additional approximation techniques or exploiting system symmetries. Floquet theory is a widely used approach, particularly suited to systems featuring Hamiltonians that are periodic in time with fixed frequencies [116]. It enables the derivation of quasi-stationary states, which represent the eigenstates of an effective, time-independent Hamiltonian referred to as the Floquet Hamiltonian. Using the Floquet–Magnus expansion or the rotating wave approximation, these states can be used to calculate several optical properties of the system, including polarizability, harmonic generation, and more. This method has also been adapted to consider different types of switching functions of external perturbations, making it incredibly versatile for the analysis and modelling of complex systems [117].

A different approach is to employ time-dependent density functional theory (TDDFT), a special case of response theory that characterizes how a system responds to external time-dependent perturbations [118,119]. TDDFT can simulate the dynamics of electronic density and effective potential under arbitrary time-dependent external potentials, and it can be implemented in various ways, such as linear response theory, nonlinear response theory, or real-time response theory. Perturbation theory is a method to approximate the solution of a problem by expanding it in powers of a small parameter, such as the magnitude of the external potential. Perturbation theory can be used in conjunction with TDDFT, for instance, to compute the linear or nonlinear response functions of a system. However, TDDFT can also capture non-perturbative phenomena, such as strong-field ionization or high-harmonic generation. Therefore, TDDFT is more general and versatile than perturbation theory.

## 4. Example: Fundamental Properties Using Density Functional Theory

At present, DFT remains the main method for investigating the chemical properties of photocatalysts. It relies on the existence of an energy functional that depends on the electron density of the system. The minimum of this functional gives the ground state energy and density [120]. Using electron density as the main variable greatly simplifies the problem compared to wave functions that require 3N coordinates, where N is the number of electrons. However, the exact form of the energy functional is unknown, and only approximate expressions are available. They are based on various assumptions about the energy functional’s dependence on electron density and might incorporate additional variables such as kinetic energy density (τ), exchange potential (ε*_x_*), and other non-local interactions. By introducing these variables, the functional becomes more adept at capturing the intricate interplay of electronic behaviors with better correctness. While it refines the functional’s developed dependence on electron density, it also poses increased computational challenges:

(9) $$E\left[\rho \right]=E\left(\underset{\mathrm{Hybrid}}{\underbrace{\left(\underset{\mathrm{GGA}}{\underbrace{\left(\underset{\mathrm{LDA}}{\underbrace{\rho }},\nabla \rho \right)}},\varepsilon_{x}\left(\varphi_{i}\right)\right)}},\ldots \right)$$

The main kinds of DFT functionals are the following (9):

- Local density approximation (LDA): This functional depends only on the local density ρ(*r*) at a given point. This is the simplest and computationally the cheapest approximation, but it is often inaccurate for systems with strong electron–electron interactions or spatial variations in density. Some examples of such functionalities are VWN [121] and PWC [122].
- Gradient-corrected approximation (GGA): This functional depends on the local density ρ(*r*) and its gradient ∇ρ(*r*). This improves the accuracy of LDA by accounting for the non-uniformity of the electron density, but it still fails to capture some important effects such as dispersion or self-interaction. The most frequently used functionals of this type are PW91 [123], BLYP [124], and PBEsol [125].
- Hybrid: This functional combines some features of ab initio methods, such as Hartree–Fock, with some features of DFT methods. This enhances the accuracy of GGA by incorporating some exact exchange and correlation effects ε*_x_*({φ*_i_*}), but it also increases the computational cost and complexity because a large number of two-electron integrals are constructed from orbitals. Examples are B3LYP [126] and PBE0 [127].
- Meta-GGA: This functional depends on the local density, its gradient, and its kinetic energy τ(*r*) density (M11-L [128], revTPSS [129]). This improves the accuracy of GGA by accounting for the non-local effects of exchange and correlation, but it also introduces new parameters and challenges for the functional design, which also increases their functionality. Additionally, such functionals, like SCAN [130], satisfy all constraints [131], which makes them favorite candidates for electronic structure calculations.
- Range-separated hybrid: This functional split the exchange and correlation into short-range and long-range components and uses different approximations for each [132]. This improves the accuracy of hybrid methods by reducing the self-interaction error and the delocalization error, but it also requires the choice of a range-separation parameter that may depend on the system.
- Double hybrid: This functional combines a hybrid functional with a perturbative correction based on MP2 or similar methods [133]. This improves the accuracy of hybrid methods by including dynamic correlation effects, but it also makes the functional very expensive and sensitive to the basis set.

The functionals are listed in order of increasing complexity and the non-locality of density dependence. In general, this leads to better accuracy, but not always. For example, LDA is simpler and faster than GGA, but GGA is more accurate and flexible for most systems. However, there are some cases where LDA performs better than GGA, such as the following:

- Elastic constants of some crystals: LDA tends to over-bind atoms and predict stiffer bonds, which results in better agreement with experimental values of elastic constants for some materials, such as diamond or silicon. GGA, on the other hand, tends to underestimate elastic constants due to softer bonds and gradient corrections.
- Phase transitions of some metals: LDA is more accurate than GGA for predicting the critical pressures of structural phase transitions of some group IV, V, and VI elements, such as C, Si, Ge, Sn, S, Se, and Te. This is because LDA is exact for a uniform gas and works better for simple metals, while GGA introduces errors due to gradient corrections and the over-delocalization of the electrons. These are some examples of where LDA is more accurate than GGA, but they are not general rules. In most cases, GGA is preferred over LDA for studying the electronic structure and properties of many-body systems. However, even GGA may not be sufficient for some systems that require more advanced functionals, such as hybrid, meta-GGA, range-separated hybrid, or double-hybrid.

**Figure 1:**
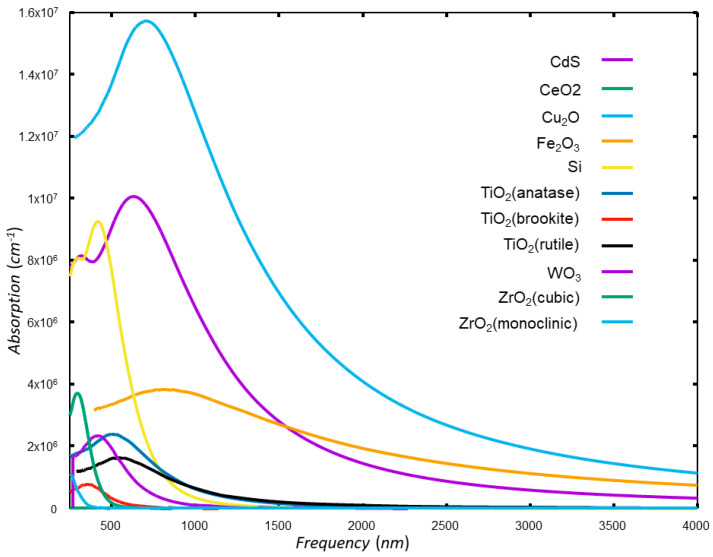
Absorption coefficients for typical photocatalysts.

**Figure 2:**
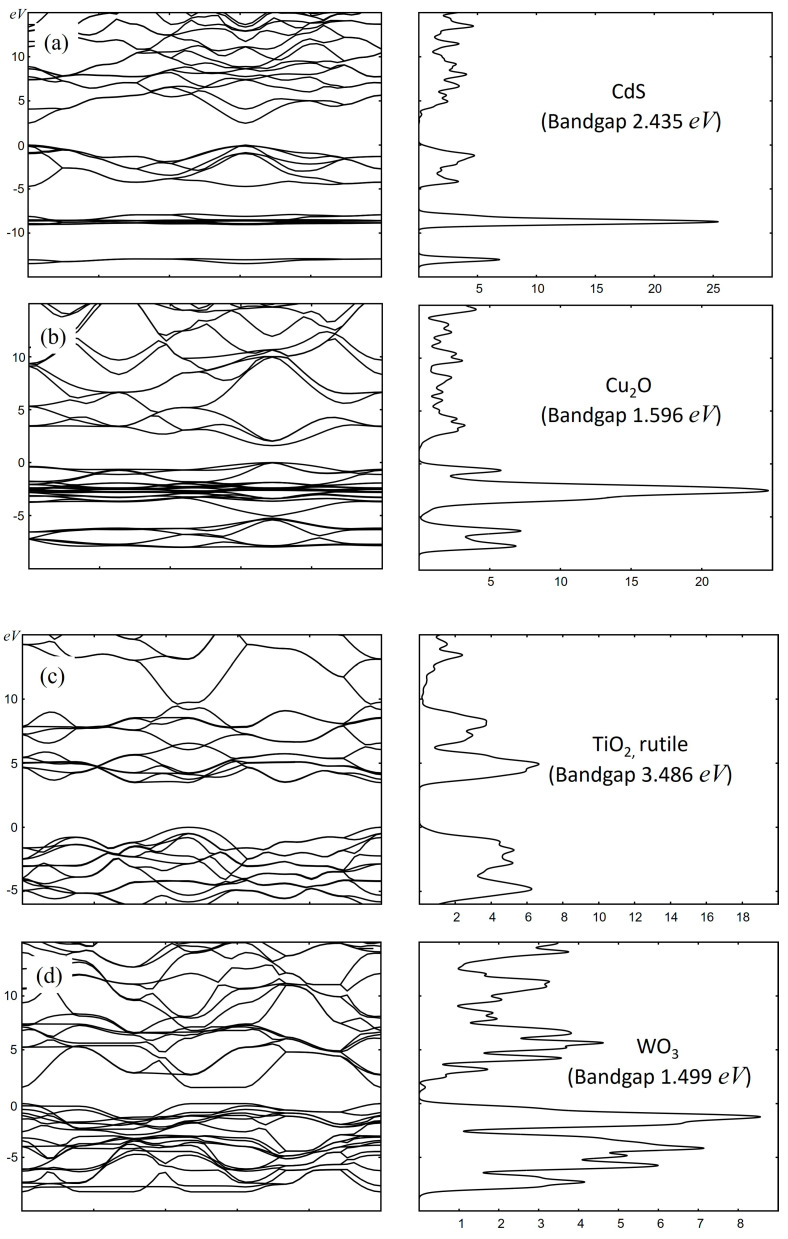
Band structures and density of states for CdS (**a**), Cu_2_O (**b**), TiO_2_ rutile (**c**), and WO_3_ (**d**).

The photocatalytic properties and performance of catalysts depend strongly on the supporting surfaces on which they are situated. In some cases, the support surface can even act as a photocatalyst itself. These surfaces can be engineered to enhance chemical efficiency by creating defects and vacancies and modifying the geometry. For instance, adjusting the HOMO–LUMO gap of a catalyst by altering the support surface can facilitate the charge separation process and improve the electron–hole transfer efficiency, thereby affecting the reaction rate [134,135]. However, such systems are too large and complicated to be explicitly modeled by DFT at the molecular level. Therefore, a specific version of DFT combined with periodic boundary conditions (PBC DFT) is employed to simulate catalysts deposited on periodic systems, such as crystals, surfaces, and nanotubes [136]. It uses Bloch’s theorem, which states that the eigenfunction of an electron in a periodic potential $\psi_{nk}\left(r\right)$ can be written as the product of a plane wave $e^{ikr}$ and a periodic function $u_{nk}\left(r\right)$. Mathematically, the Bloch theorem can be expressed as follows:

(10) $$\psi_{nk}\left(r\right)=e^{ikr}u_{nk}\left(r\right)$$

where *n* is the band index, and *k* is the wave vector [137]. PBC DFT solves the Schrödinger or, more precisely, Kohn–Sham equation for the Bloch wave function using a self-consistent approach that involves the electron density, the exchange-correlation energy, and the effective potential. PBC DFT can compute the energy band structure, the density of states, optical properties (reflectivity, absorption, refractive index, dielectric function…), and other electronic properties of periodic materials. This method not only lowers the computational cost but also enables the investigation of the electronic structure and other essential properties of periodic systems, offering valuable insights into the photocatalytic behavior of materials, especially those supported on complex surfaces. In Figure 1 and Figure 2, examples of calculated absorption curves and electronic properties (the band structure and density of states) of several common catalytic materials (CdS, CeO_2_, Fe_2_O_3_, Si, TiO_2_, WO_3_, ZrO_2_) are presented. These calculations were conducted using the HSE06 density functional theory (DFT) functional as it is implemented in the CASTEP software package, (version 24.1, Castep Developers Group, UK).

Due to periodic symmetry, using plane wave basis sets is more appropriate for PBC DFT [138]. This symmetry implies that the potential energy and the electron density are invariant under a discrete set of lattice translations, which allows us to define a primitive unit cell that can be repeated infinitely in all directions to form the crystal. The wavefunctions of the electrons in the crystal can then be written as Bloch functions, which are the product of a plane wave and a periodic function with the same periodicity as the crystal. This simplifies the computation of electronic properties, such as the band structure and the density of states, by reducing the problem to a finite number of k-points in the Brillouin zone. One can also reduce the computational cost by using pseudopotentials to replace the atomic cores and account for their effect on the valence electrons. Pseudopotentials are effective potentials that smooth out the oscillations of the wavefunctions near the nuclei and remove the core electrons from the simulation. This avoids the singularities of the Coulomb potential and reduces the number of plane waves needed to represent the wavefunctions accurately. Pseudopotentials also decrease the size of the basis set needed for full system convergence, which lowers the computational effort for PBC DFT calculations significantly.

Unfortunately, in addition to the computational cost and the basis set dependence, there is an additional difficulty in using hybrid DFT functionals for periodic systems associated with the treatment of long-range interactions. Hybrid functionals are intended to improve the treatment of long-range electrostatics. However, the non-local character of these contributions means that they can be computationally expensive and difficult to apply accurately in periodic systems [139]. Also, they often fail to describe dispersion forces, which are important for weakly bound systems such as molecular crystals, van der Waals solids, and layered materials. To account for these effects, one may need to use empirical corrections or more sophisticated methods such as range-separated hybrids or self-interaction corrections. However, these approaches also introduce new parameters and approximations that may affect the accuracy and transferability of the results. Therefore, choosing the appropriate hybrid functional for periodic systems is not a trivial task and requires careful validation and comparison with experimental data or higher-level methods.

To model a semi-infinite slab using PBC DFT, a primitive unit cell of the bulk crystal should be employed and then cut along a desired surface plane to create the slab. The thickness of the resulting slab should be carefully chosen to ensure the avoidance of artificial interactions between the top and bottom surfaces. Furthermore, a vacuum layer should be included within the system to help separate the slab from its periodic images along the direction normal to the surface. The thickness of the vacuum layer should be appropriate to prevent spurious interactions between the slabs while avoiding excessive computational costs.

A typical approach to model the photocatalytic process on the surface involves different modeling strategies, ranging from the simple assumption that the surface atoms are arranged in the same way as the bulk atoms, and that the surface energy is proportional to the number of dangling bonds, to the consideration of the rearrangement and relaxation of the surface atomic positions due to the lower coordination number and higher reactivity of the surface atoms, as well as the introduction of various types of defects on the surface, such as vacancies, adatoms, steps, kinks, and impurities, and the interaction between the surface and the adsorbed molecules or atoms, such as water, oxygen, hydrogen, and hydroxyl groups. The latter can mimic the realistic photocatalytic environment, but it may involve multiple adsorption sites and configurations that need to be optimized [140].

Today, DFT is successfully applied in the electronic structure analysis of photocatalysts (see Section 3) to study the location of important orbitals, the redistribution of electronic density due to excitation, the creation of an electron–hole pair(s) and following reduction reactions during photocatalysis, DOS and band structure, etc. Researchers use DFT for materials screening and design, making DFT an essential tool in advancing our understanding of photocatalysis and sustainable energy conversion. This type of calculation has been the subject of much research and has also been reviewed in various publications showing the importance of DFT calculations. For example, Butera [141] provides practical guidance for researchers in the field of both homogeneous and heterogeneous catalysis, covering atomic-centered basis sets, plane waves, and energy barrier evaluation. The work discusses two concepts used to understand the kinetics of chemical reactions, particularly in catalysis: transition state theory (TST) and the energetic span model (ESM). TST describes how chemical reactions occur and how reaction rates are determined and assumes that the rate of the reaction is proportional to the concentration of the transition state. On the other hand, the newer ESM concept focuses on the energy differences between intermediates and transition states in a catalytic cycle and defines the turnover frequency (TOF) of a catalytic event by identifying the energetic span of the cycle. The ESM helps in understanding the maximum turnover and the degree of turnover frequency control for states in each reaction pathway. The application of density functional theory (DFT) methods to catalysis is discussed, which serves as a practical guide for using DFT in both homogeneous and heterogeneous catalysis, providing insights into the selection of catalytic models and the evaluation of energy barriers.

Another recent study [142] explores the multifunctional properties of XO2 monolayers (where X = Ti, Ni, Ge) using DFT calculations. Researchers delve into structural, electronic, optical, and photocatalytic aspects, shedding light on the exciting potential of these materials and exploring the effects of strain and stacking on these monolayers. The study provides valuable insights into the properties of these monolayers and their potential applications in various fields. In the work presented by Wenzhi Yao [143], the g-C3N4/BiOBr (001) heterostructure was analyzed through DFT calculations to understand its geometric and electronic structures and how they contribute to its photocatalytic abilities. The study reveals that the heterojunction functions as a type-II heterojunction, which facilitates effective electron–hole separation at the interface. Additionally, it is found that applying an external electric field can tune the electronic structure and enhance the optical absorption in the visible region, potentially improving photocatalytic performance. These insights could lead to advancements in photocatalysis technology, particularly in applications like solar energy utilization and pollutant degradation.

However, conventional descriptors, such as HOMO–LUMO, (p)DOS, and band structure, may not always be adequate to fully characterize the electronic structure of photocatalysts. These descriptors can be limited, particularly when studying systems with strong electron correlation or when focusing on excited states [1,144]. Therefore, utilizing more advanced techniques may be necessary to overcome these limitations and accurately investigate the electronic properties of photocatalysts.

For example, when dealing with strongly correlated molecular systems, such as in transition metal complexes and rare earth compounds, single determinant approximations such as DFT may fail [145], and the energy gap between frontier orbitals might not be sufficient to study the system’s properties accurately. Instead, multi-reference methods should be used, in which the concept of separate orbitals loses its meaning. The focus shifts to the wave function itself, from which chemically representable constructs like orbitals can be derived through post-processing to yield natural orbitals when needed. For the analysis of excited states and electronic density redistribution, Dyson and Brueckner orbitals offer valuable insights [146]. Dyson orbitals are obtained by solving the Dyson equation and are used to describe the excited states of a molecule. In contrast, Brueckner orbitals result from the maximum overlap between the Slater determinant and the wavefunction of many electrons [147]. Dyson orbitals are used to describe single-electron approximations for excited states of a molecule, while Brueckner orbitals are used to calculate the ground-state properties of a molecule by correcting for electron–electron repulsion.

Investigating the nonlinear optical properties of materials through their response to electric fields yields vital insights for photocatalysis [148]. Such studies are key to determining the effectiveness of light absorption and charge transfer processes. For instance, the absorption of two photons is a crucial nonlinear optical phenomenon that has been extensively studied in both organic and inorganic materials. Its discovery has led to the development of materials with enhanced optical properties. In photochemistry, this phenomenon is associated with the absorption of light in the visible region of the electromagnetic spectrum, making it a highly relevant area of study. Many organic molecules exhibiting significant nonlinear optical activity have been utilized as photocatalysts [149,150]. By focusing on linear and non-linear responses, researchers can predict material interactions with light and their role in facilitating charge carrier dynamics. This research is crucial for advancing photocatalytic materials designed for environmental applications, including water splitting and CO_2_ reduction. Moreover, theoretical models that calculate these responses help tailor photocatalysts’ electronic structures, enhancing their light reactivity. This leads to the development of photocatalysts with broader light spectrum capabilities, improved stability, and better performance, which are essential for real-world applications.

To study the specific reactivity of a molecule, the Fukui function can be efficiently utilized, as it describes the change in electron density at a given point in the molecule when an electron is added or removed [151]. This information provides valuable insights into the reactivity of different sites within a molecule during chemical reactions, allowing for the identification of reactive sites by highlighting where electrons are likely to be accepted or donated. In the context of photocatalysis, this predictive capability is critical in forecasting the interactions between a photocatalyst, light, and other reactants. Precise information on such interactions is essential for refining processes such as water splitting or carbon dioxide reduction. Analyzing Fukui functions, for instance, can enhance our understanding and control of photocatalytic reactions by substantiating the underlying mechanisms [152]. Even more accurate information could be extracted from the dual descriptor [153]; while the Fukui function can reveal the nucleophilic and electrophilic regions on a molecule, the dual descriptor can unambiguously expose truly nucleophilic and electrophilic regions with greater reliability. Moreover, the dual descriptor is less affected by the lack of relaxation terms than the Fukui function when the frontier molecular orbital approximation is used. As a result, the dual descriptor can be considered a more trustworthy descriptor for measuring local reactivity than the Fukui function [154].

**Figure 3:**
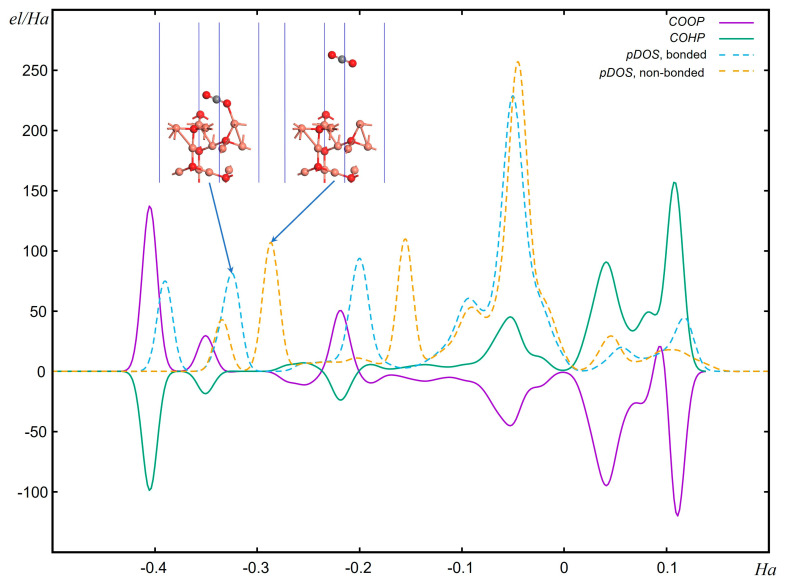
Crystal orbital overlap and Hamilton populations for carbon and copper atoms of CO_2_ molecule bonded to a Cu_2_O(111) surface. The partial DOS is also included for both the bonded and nonbonded systems.

To gain insights into chemical bonding, the analysis of crystal orbital overlap population is a powerful tool [155]. In the context of photocatalysis, the orbital overlap population is a measure of the electron density shared between overlapping orbitals, which is indicative of the strength of bonding interactions between atoms. Predominantly, the crystal orbital overlap population (COOP) [156] and the crystal orbital Hamilton population (COHP) [157] stand as pivotal methodologies in the quantum chemical analysis of bonding phenomena in solid-state systems, but they analyze different aspects. COOP divides the electron density between atoms, indicating whether interactions are bonding or antibonding. Integrating a COOP curve yields the electron count, akin to the Mulliken population analysis. On the other hand, COHP dissects the band structure energy into bonding and antibonding contributions. Integrating a COHP curve offers an energy value that reflects the bond’s strength. Thus, while COOP informs us about electron distribution, COHP reveals the energetic implications of bonding within the crystal. Both analyses are valuable for studying photocatalysis, as they offer insights into the electronic structure and stability of the catalysts involved in light-induced chemical reactions. Understanding these interactions can help optimize the design and efficiency of photocatalytic materials. In practical photocatalytic studies, understanding the orbital overlap population can provide insights into the electronic structure of the catalysts and the nature of the interactions between the catalyst and reactants [158]. This information is crucial for modeling the processes involved in photocatalysis, such as light absorption, charge separation, and transfer, as well as the reactivity of the catalyst. By analyzing the orbital overlap population, researchers can predict and optimize the efficiency of photocatalytic reactions, leading to the development of better photocatalysts for applications like water splitting and CO_2_ reduction. Figure 3 illustrates an example of such an analysis for carbon and copper atoms in the case where a carbon dioxide molecule is bonded to the (111) surface of a copper oxide. The partial DOS would show the contribution of these atoms to the electronic states of the system. In a bonded system, one would expect shifts in the pDOS due to the interaction between CO_2_ and the Cu_2_O surface, indicating charge transfer and bonding characteristics. For CO_2_ bonded to Cu_2_O (111), the analysis suggests that CO_2_ can bind as a tilted linear molecule coordinated to an unsaturated surface cation, and the presence of surface vacancies can influence the adsorption and activation of the CO_2_ molecule. These interactions would be reflected in the COOP and COHP analyses, as well as in the partial DOS for the carbon and copper atoms.

In photocatalysis, precise energies and correct electronic density redistributions are required, and this can sometimes exceed the capabilities of even the most advanced density functional theory. In such instances, turning to multireference methods and many-body perturbation theory [159,160] can offer a significant enhancement in the quality of modeling photocatalytic reactions. These methods are adept at capturing the complex electronic interactions often present in photocatalytic systems, particularly when dealing with excited states or systems where electron correlation is non-negligible. By incorporating multireference approaches, researchers can achieve a more nuanced and comprehensive understanding of photocatalytic processes, potentially leading to breakthroughs in the efficiency and effectiveness of photocatalysts.

## 5. Software

The landscape of quantum chemistry software packages is exceptionally diverse, accommodating an array of commercial and free software options tailored to various molecular and periodic boundary conditions, density functional theory, and wave function-based applications. Depending on the research objectives, different software packages may offer different advantages (and also disadvantages) in terms of methodology, parallelization, and accessibility. In this brief review, we will briefly introduce some of the popular and widely used quantum chemistry software packages and highlight their main features and capabilities.

For molecular quantum chemistry, one of the most comprehensive and multipurpose software packages is Gaussian 16 (Gaussian Inc., Wallingford, CT, USA) [161], which is a commercial software that can perform a wide range of molecular methods, from HF and DFT to multiconfigurational models, such as CASSCF and MRCI. Gaussian also has an extensive library of density functionals, including local, gradient, hybrid, meta, and range-separated, as well as various basis sets, such as Gaussian-type orbitals (GTOs) and effective core potentials (ECPs). G16 can also calculate excited states with different approaches, transition states, and reaction paths using methods such as configuration interaction singles (CIS), TDDFT, and intrinsic reaction coordinates (IRC). Moreover, it offers the possibility of fine-tuning a number of parameters, such as convergence criteria, integral accuracy, and memory allocation, as well as providing an additional output, making it an excellent tool for advanced simulations and the development. G16′s parallelization capabilities are highly optimized for symmetric multiprocessing (SMP) workstations and single cluster nodes, although arranging it across multiple cluster nodes necessitates the Linda parallel library, which is an additional cost.

Another famous commercial software for molecular quantum chemistry is Amsterdam Density Functional (ADF SCM Inc., Amsterdam, The Netherlands) [162], which specializes in molecular DFT, emphasizing Slater determinants and optimization strategies for efficient parallelization. The ADF can perform various DFT methods, such as generalized gradient approximation (GGA), hybrid, meta-GGA, and range-separated functionals, as well as relativistic and spin–orbit coupling effects. ADF can also calculate excited states, transition states, and reaction paths using methods such as TDDFT, constrained DFT (CDFT), and transition state search (TSS). Additionally, ADF can handle large and complex systems, such as transition metal complexes, organometallics, and biomolecules, using methods such as frozen core approximation, fragment molecular orbital (FMO), QM/MM (quantum mechanics/molecular mechanics), and ONIOM.

A free-for-academia, general purpose software that rivals G16 and ADF in its functionalities is Orca (version 5, Max Planck Institute, Germany) [163,164], which prioritizes coupled cluster, multi-reference, semi-empirical, and DFT methods. Orca can perform various coupled cluster methods, such as CCSD (CC with singles and doubles), CCSD(T) (CC with singles, doubles, and perturbative triples), and CCSDT (CC with singles, doubles, and triples), as well as various multi-reference methods, such as CASSCF, MRCI with density matrix renormalization group (DMRG), and the resolution of identity approximations; so, it offers advanced scalability and optimization, making it an excellent choice for complex quantum chemistry simulations. Orca can also perform various DFT functionals, such as GGA, hybrid, meta-GGA, and range-separated functionals, as well as various GTO basis sets and ECPs. It can also calculate excited and transition states and reaction paths using methods such as CIS, TDDFT, and IRC.

For PBC quantum chemistry, one of the industry leaders is Vienna Ab initio Simulation Package (VASP, version 6, University of Vienna) [165], which is a commercial software that can perform PBC DFT and many-body perturbation theory methods, such as GW approximation, the Bethe–Salpeter equation (BSE), and random phase approximation (RPA). VASP can perform various DFT approaches, such as GGA, hybrid, meta-GGA, and range-separated functionals, as well as various basis sets, such as plane waves, projector augmented waves (PAWs), and ultrasoft pseudopotentials (USPPs). Moreover, VASP excels in flexibility concerning functional and basis set selection, including commercial parametrizations, such as PBE0, HSE06, and PBEsol.

Quantum Espresso (version 7, Quantum Espresso Foundation) [166], on the other hand, offers a wide range of functionalities, and its open-source code can be shared and modified by researchers. As a result, it can benefit from new developments and innovations very quickly. Quantum Espresso can perform various DFT functionals, as well as various basis sets, such as plane waves, PAWs, and norm-conserving pseudopotentials (NCPPs). Quantum Espresso can also perform various many-body perturbation theory methods, such as GW approximation, BSE, and RPA. As a typical PBC DFT software, Quantum Espresso can also calculate band structures, the density of states, optical properties, and surface reactions using methods such as k-point sampling; in addition, it could be combined with Wannier functions, dielectric functions, and NEB. Additionally, Quantum Espresso can handle large and complex systems, such as nanomaterials, biomolecules, and interfaces, using methods such as FMO, QM/MM, and embedded cluster model (ECM).

The CASTEP (version 24, Castep Developers Group, led by Dr. Matthew Segall) package [167,168] is a powerful tool for computational materials science that comes embedded in the commercial suite Material Studio and is also available free of charge for academic use upon request. In addition to typically use in PBC LDA and GGA functionals, it offers a comprehensive range of exchange-correlation functionals, including hybrid (PBE0, B3LYP, HSE03, HSE06) and meta-GGA (RSCAN), as well as Hartree–Fock. In addition, CASTEP employs a highly efficient plane-wave basis set and supports both ultrasoft and norm-conserving pseudopotentials. A significant advantage of CASTEP is that it has a built-in “on the fly” (OTFG) pseudopotential generator that enables users to create customized pseudopotentials for any element without dependence on external databases. The package is also highly flexible, allowing for different optimizations of unit cell and atom positions. Also, it has efficient parallelization via the use of OpenMP and MPI, enabling large-scale calculations with reduced computational time. However, CASTEP does not include many-body perturbation methods such as GW and the Bethe–Salpeter Equation (BSE), which limits its applicability to certain classes of materials systems, but it provides a wide range of spectroscopic features, including IR and Raman spectra, core level spectra, and NMR, which are directly related to the experiment.

DMol3 (version 2024, BIOVIA Inc., San Diego, CA, USA) [169] is a versatile software package that enables density functional theory (DFT) calculations on a wide range of periodic systems including molecules, clusters, surfaces, and solids. Contrasting with other programs, DMol3 employs a numerical radial function basis set (with the possibility to add scalar relativistic corrections) that is incredibly efficient and space-saving compared to the plane-wave basis set used in packages like CASTEP. Furthermore, DMol3 supports the conductor-like screening model (COSMO) to accommodate solvation effects and enable the simulation of solvated molecules and wet surfaces. With greatly enhanced MPI/OpenMP parallelization, DMol3 can efficiently handle even large systems, making it an excellent choice for complicated calculations. Additionally, it calculates electron transport properties, such as transmission and current, using non-equilibrium Green’s function theory.

The OpenMX package (version 2023, Northwestern University, Illinois) [170] is a highly advanced computational tool that utilizes a versatile linear combination of pseudo-atomic orbitals (LCPAO) basis set, which provides greater flexibility and transferability compared to other commonly used basis sets such as plane-wave or numerical radial function basis sets. The package offers a range of robust features including the capacity to handle spin–orbit coupling, non-collinear magnetism, spin-polarized DFT+U, spin Hall effect, and non-equilibrium Green’s function calculations. Moreover, the tool is executed efficiently through its well-parallelized implementation in both OpenMP and MPI parallelization models. As an open-source software, OpenMX is effectively configured for efficient compilation with a wide range of compilers, such as GNU and Intel, and numerical libraries like MKL, ELPA, among others.

OpenMolcas (version 23, Lund University, Sweden) [171] should also be noted as a convenient tool for new developments in quantum chemistry. It is a general-purpose software package that specializes in multiconfigurational methods, making it an excellent choice for accurately describing the electronic structure of molecules. With its core features, such as CASSCF and CASPT2, OpenMolcas is well-equipped to handle complex electron correlation issues. Also, its ability to predict spectral properties makes it ideal for investigating the electronic and optical properties of molecules in detail. As an open-source platform, it promotes collaborative development and the integration of new features and improvements by the scientific community. The software’s architecture is deliberately designed to simplify the integration of novel developments, fostering innovation and facilitating the exploration of new concepts within the realm of multiconfigurational methods.

DFTB+ (version 24) [172] is regarded as the most extensively used software implementation of the DFTB approach available as a standalone free program and also integrated into computational chemistry software packages (Material Studio, ADF, and Atomistix Toolkit). It employs a minimal basis set of valence orbitals and a parametrized Hamiltonian based on the Slater–Koster model that provides an ideal balance between speed and accuracy, and it is widely used. DFTB+ can handle various DFTB methods, corrections, and spin effects and can perform electronic structure calculations for large and complex systems. Additionally, it can undertake time-dependent DFTB, excited states, and transport calculations, including various advanced extensions, such as LDA+U, spin–orbit coupling, and pseudo self-interaction correction, which significantly enhance the precision and predictability of the outcomes. The program allows for the calculation of various electronic properties, e.g., band structures, density of states, optical spectra, and surface reactions, using state-of-the-art techniques such as k-point sampling, Wannier functions, dielectric functions, the nudged elastic band (NEB) method, and the particle–particle random-phase approximation (pp-RPA). These methods provide scientific insights into the electronic behavior and reactivity of the system, as well as its optical and transport properties. Moreover, DFTB+ permits computations of optimization, frequency, dynamics, and meta-dynamics, which further diversifies its range of applications and computational performance. The efficient parallelization and diagonalization of DFTB+ using MPI and OpenMP (and GPU) give it a competitive edge in computational chemistry.

## 6. Conclusions and Perspectives

Computational modeling in photocatalysis has emerged as a powerful and influential tool in various fields of application, such as energy and material science, over the past few decades. This is related to general progress in computational and quantum chemistry. However, to keep up with the advances in experimental techniques and industrial needs, modeling requires continuous and significant extensions of its capabilities. This could be achieved through five factors:

1. The further development of quantum chemical algorithms;
2. The development of numerical algorithms;
3. The exploitation of the structure of molecular systems;
4. Parallelization and new computational hardware;
5. New paradigms.

Analyzing the first three items, we can conclude that none of them alone can provide sufficient improvements to meet modern needs. For example, most quantum chemical methods scale polynomially with the size of the system, meaning that simulating a photocatalyst bonded to a nanoparticle, which is orders of magnitude larger than a medium-size molecule, would entail a substantial increase in computational demand, which is not matched by the new hardware that mostly follows Moore’s law. On the other hand, the development of numerical techniques and the use of molecular system structure (such as clustering) aim at linearization, which is good but not good enough, because one needs orders of magnitude faster methods. Also, one needs to consider that most computational chemistry methods have several bottlenecks (such as the calculation of integrals, matrix diagonalization, etc.), and improving one part of the method could reveal other problems that were not seen before. Next, parallelization, with both traditional and new GPU units, also faces challenges, because with an increased number of processing units, the overhead grows, and almost all modern techniques do not scale linearly with the number of processors, as stated by Amdahl’s law. Based on all of that, we can say that only the combination of 1–4 could address the problem of increasing demands for computational modeling in the photocatalysis field. This combination naturally leads to the multiscale concept, which involves the construction and solution of models that combine “sub-models” of multiple scales of the target system, to achieve superior modeling results or higher computational efficiencies, which can hardly be achieved by single-scale models. Multiscale modeling has existed for many years in basic science and engineering areas, such as mathematics, material science, chemistry, and fluid dynamics. There are now a few very successful multiscale methods that enable a dramatic improvement in the applicability of traditional single scale methods (ONIOM, QM/MM, …).

Unfortunately, the development of the multiscale approach grew slowly because there is no general theory of multiscale. There are some attempts to construct a mathematical basis for it, but it is still in the early stages, because this development requires expertise in many fields and researchers typically focus on their particular tasks rather than general theory development. Also, there is no generally accepted scientific language for the description of multiscale models and methods, and researchers working on developing multiscale methods in different fields cannot communicate effectively with each other. Different disciplines may have different definitions and interpretations of these concepts, depending on the nature of the phenomena they study. For example, in physics, scale may refer to the length, time, or energy scales of a system, while in biology, scale may refer to the level of organization, such as molecular, cellular, tissue, organ, or organism. Resolution may refer to the degree of detail or accuracy of a model or a measurement, which may depend on the available computational or experimental resources. A multiscale model or method should be able to capture the essential features of a system at different scales and resolutions and to link them in a consistent and coherent way.

Another challenge of developing a general theory is to establish criteria and methods for validating and verifying multiscale models and methods. Validation is the process of checking whether a model or a method agrees with empirical data or real-world phenomena, while verification is the process of checking whether a model or a method is implemented correctly and consistently. Both validation and verification are essential for ensuring the reliability and credibility of multiscale models and methods, but they are also difficult and complex, because they involve multiple sources of uncertainty and error, such as model assumptions, parameter estimation, numerical approximation, measurement error, and data quality. A general theory of multiscale should provide a systematic and rigorous framework for addressing these issues and evaluating the performance and limitations of multiscale models and methods.

Regarding the use of new paradigms in quantum chemistry, we have two main directions: machine learning and artificial intelligence (ML/AI) and quantum computers (QC). ML/AI can enhance quantum chemistry by providing efficient and accurate methods for data analysis, model development, and prediction. For example, ML/AI can help design new molecules, optimize reaction conditions, and discover new chemical phenomena. QC can enable quantum chemistry by offering novel and powerful ways to simulate quantum systems and solve complex problems. For example, QC can help calculate molecular properties, explore potential energy surfaces, and perform quantum dynamics. Both ML/AI and QC have the potential to revolutionize quantum chemistry by expanding its scope, accuracy, and efficiency. However, they also face significant challenges, such as data quality, algorithm development, hardware limitations, and error correction.

Machine learning is extensively used in different aspects of quantum chemistry. Mainly, there are two elements: first, using ML to improve existing quantum chemical methods; and second, using ML/AI to directly make predictions based on existing data.

The first application of ML is to enhance the accuracy and efficiency of quantum chemical methods, such as DFT, CC, quantum Monte Carlo (QMC), and multiconfigurational approaches (e.g., CASSCF or MRCI). For example, ML can help design new exchange-correlation functionals for DFT, which are crucial for capturing the electronic structure and properties of molecules and materials. ML can also help reduce the computational cost of high-level quantum methods, such as CC and QMC, by using low-level methods, such as Hartree–Fock (HF) and DFT, as reference points and correcting errors with ML models. ML can also help improve the convergence and stability of quantum methods, such as self-consistent field (SCF) iterations and geometry optimizations, by using ML models to guide the search for optimal solutions.

The second application of ML is to bypass quantum chemical methods and directly predict the properties and behaviors of molecules and materials from existing data. For example, ML can help construct interatomic potentials, which are functions that describe the interactions between atoms, and use them to perform molecular dynamics simulations, which are numerical methods that model the motions of atoms and molecules. ML can also help predict various molecular properties, such as energies, forces, dipole moments, polarizabilities, and spectra, from molecular descriptors, such as atomic coordinates, chemical compositions, and molecular graphs. ML can also help discover new chemical phenomena, such as reaction pathways, catalytic mechanisms, and phase transitions, by analyzing large and complex data sets from experiments or simulations.

A promising application of machine learning in photocatalysis properties prediction is the use of chemical descriptors derived from quantum chemistry calculations to characterize the catalytic activity of materials. ML frameworks have demonstrated remarkable capabilities for predicting material properties by establishing a nonlinear map between input and output data that correspond to the properties of interest of catalytic materials. Unlike traditional computational methods that depend on computationally intensive quantum chemical calculations, ML methods can offer fast and cost-efficient predictions of catalytic properties. ML models are trained using flexible algorithms, but the success of such models relies on the design of descriptors that can uniquely represent materials, be easily computed at low computational costs, and reflect the nature of the targeted properties. To address this, researchers have devised descriptors such as local geometric features, individual atomic features of potential active sites, and generalized coordination descriptors such as valence, free bonds, and ionic radius. However, modeling catalytic reactions requires following reaction pathways, which is a very challenging and tedious task in computational chemistry. Although atomic fingerprints can be effectively used to predict the physical properties of bulk materials, such as thermodynamics, viscosity, boiling point, fracture toughness, and density, they are inadequate for catalytic reaction path modeling. Therefore, ongoing research focused on developing new catalytic descriptors and exploring their use with ML approaches is essential for the continued advancement of this field.

One of the main challenges of applying ML in computational chemistry is that it cannot provide an understanding and explanation of the results it produces. ML models are often regarded as black boxes, meaning that their internal workings and logic are not transparent or interpretable to users. This poses a problem for computational chemistry, where the goal is not only to predict the outcomes of chemical phenomena but also to understand the underlying mechanisms and principles that govern them. For example, ML models can accurately predict the reaction rates, selectivity, and pathways of catalytic reactions, but they cannot explain why certain catalysts are more active or selective than others or what the key factors are that influence the reaction mechanisms. This limits the applicability and usefulness of ML models for the rational design and optimization of catalysts and reactions.

Another solution that has the potential to revolutionize computational chemistry and, in particular, the modeling of photocatalytic reactions is the use of quantum computations [173]. Initially proposed by Richard Feynman to solve complex Schrödinger equations for large and strongly correlated systems, quantum computing now holds potential for improving multiple scientific fields, including artificial intelligence, machine learning, and cryptography. Remarkable progress in quantum computing has resulted in the development of new and innovative quantum algorithms that have led to the accurate prediction of the electronic structure of molecules, such as the qubit coupled cluster method [174], the multireference quantum Krylov algorithm for strongly correlated electrons [175], and the quantum imaginary time evolution algorithm [176]. Quantum computing has also facilitated the simulation of chemical dynamics [177,178] with promising precision and computational speed. Furthermore, quantum computing has already exhibited practical effectiveness in fields such as determining the excited state energies of small molecular systems [179], simulating molecular electronics [180], and tracking Diels–Alder reaction pathways [181]. Researchers have also used a quantum computer to design new catalysts for CO_2_ reduction, which is an important reaction for mitigating climate change and producing renewable fuels.

Quantum computers also confront substantial challenges in terms of scalability, noise, error correction, and difficulties with new algorithms development. Current quantum computing hardware has a restricted number of qubits, which in turn limits the size and complexity of the molecular systems and processes that can be emulated. Moreover, high noise and error rates are common among current quantum computers, which negatively affects the accuracy and reliability of outcomes. In addition, efficient and robust error-correction schemes are required, which consume a significant number of qubits and resources. The demand for robust error-correction schemes is driving the development of more efficient quantum resource management. The need for novel algorithms is not a hurdle but an opportunity to harness the full potential of quantum advantages. This exciting phase in quantum computing is fostering interdisciplinary collaboration and inspiring breakthroughs in photocatalysis and chemistry, heralding a future of limitless possibilities.

This short review comprehensively discusses current quantum chemistry methods for modeling photocatalysts. To more effectively illustrate the main attributes of current quantum chemistry approaches employed in photocatalysis modeling, Table 1 is provided, thus allowing for a simple comparison of their strengths and weaknesses. Special attention is paid to molecular and periodic boundary condition (PBC) density functional theory (DFT) approaches, which have shown particular success in various photocatalytic systems such as metal oxides, metal chalcogenides, carbon-based materials, metal halide perovskites, and metal–organic frameworks. The advantages and limitations of these methods are presented, with practical applications and examples. In addition, the most widely used computational chemistry software packages are carefully reviewed and compared, considering their features, performance, and accuracy. Practical tips are included to aid researchers in selecting the best software for their specific research problem. Finally, this review addresses the challenges and perspectives for the future development of quantum chemistry methods for modeling photocatalysts, focusing on the need for a general multiscale theory capable of bridging different length and time scales. Additionally, this review considers the potential of machine learning techniques to accelerate the discovery and optimization of novel photocatalysts and the promise of quantum algorithms to overcome the limitations of classical computers. This review is intended to serve as a valuable reference and guide for researchers and students interested in the field of photocatalysis and its computational modelling.

## Figures and Tables

**Table 1:** Comparative analysis of methods regarding photocatalytic systems.

Method	Description	Strengths	Limitations	Extensions	Applications in Photocatalysis
Density functional theory (DFT)	Minimizes functional of electronic density, for which only approximate expressions are currently available.	Widely used for its efficiency and good balance between accuracy and computational cost.	May struggle with systems that have strong correlation effects or require accurate description of excited states.	New functionals (SCAN, Double hybrid, range separated, …), CASDFT, density fitting	Geometry optimization, orbital energies, TS, reaction barriers, DOS, band structure
Multiconfigurational and multireference methods (MRCI, CASSCF, RASSCF …)	Consider multi configurational states for a more accurate description of systems with significant electron correlation.	Provide a better treatment of systems with near-degeneracy and strong static correlation.	Computationally intensive and may not be suitable for very large systems.	Combination with DFT (CASDFT) and AI/ML, DMRG, resolution of identity,	Excitations, spectroscopy, accurate electronic density distributions
Perturbation theory (MP2, MBPT)	Account for electron correlation beyond the mean-field approximation by using perturbative corrections.	Can accurately describe quasiparticle excitations and excited states.	Requires high computational resources and expertise to apply correctly.	New GW and Green function approximations, combination with CAS (CASPT2)	Excitations, accurate adsorption energies, quasiparticle, and exciton binding modeling
Semiempirical methods (MNDO, AM3, PM6, PM7, DFTB, …)	Utilize empirical parameters obtained from experimental data or high-level calculations to account for electron correlation effects	Less computationally demanding, suitable for large systems and long time-scale simulations	Less accurate than ab initio methods; may not capture all relevant physical interactions; needs parametrization	New Hamiltonians, combinations with more accurate methods in QM/MM, ONIOM, …	Preliminary geometry optimization, modeling large systems, properties

## Data Availability

The study did not report any data.
